# Who Sets the Global Health Research Agenda? The Challenge of Multi-Bi Financing

**DOI:** 10.1371/journal.pmed.1001312

**Published:** 2012-09-25

**Authors:** Devi Sridhar

**Affiliations:** Blavatnik School of Government & Department of Politics and International Relations, University of Oxford, Oxford, United Kingdom

## Abstract

As part of a cluster of articles critically reflecting on the theme of “no health without research,” Devi Sridhar discusses a major challenge in the governance of research funding: “multi-bi” financing that allows the priorities of funding bodies to dictate what health issues and diseases are studied.

**Summary Points**

- A major challenge in the governance of research funding is agenda-setting, given that the priorities of funding bodies largely dictate what health issues and diseases are studied.
- The challenge of agenda-setting is a consequence of a larger phenomenon in global health—“multi-bi financing.”
- Multi-bi financing refers to the practice of donors choosing to route non-core funding—earmarked for specific sectors, themes, countries, or regions—through multilateral agencies such as the World Health Organization (WHO) and the World Bank and to the emergence of new multistakeholder initiatives such as the Global Fund to Fight AIDS, Tuberculosis and Malaria and the GAVI Alliance.
- These new multistakeholder initiatives have five distinct characteristics: a wider set of stakeholders that include non-state institutions, narrower problem-based mandates, financing based on voluntary contributions, no country presence, and legitimacy based on effectiveness, not process.
- The shift to multi-bi financing likely reflects a desire by participating governments, and others, to control international agencies more tightly.

*In anticipation of the 2012* World Health Report, *this paper was commissioned to help contextualize and critically reflect on the theme of “no health without research.”*

## Introduction

A major challenge in the governance of research funding is priority-setting. As a former health minister in sub-Saharan Africa noted, “Everyone is chasing the money—reputable universities, the UN agencies, partnerships, civil society groups, so who is actually doing what developing countries really need, rather than what donors want?” [1] The past 15 years have been called revolutionary in global health in terms of the funding raised and the number of initiatives launched. One of the side effects of having more money, institutions, and initiatives in global health is increased competition among the various parties. And, the priorities of funding bodies largely dictate what health issues and diseases are studied.

In this Essay, I argue that the challenge of agenda-setting that occurs in research funding is a consequence of a larger phenomenon in global health, “multi-bi financing.” Multilateral funding refers to monies given to an organization that involves two or more governments or other institutions, the prime example being the United Nations; bilateral funding refers to monies given from one government or institution to another such as the US Agency for International Development (USAID) grants to Haiti. Multi-bi financing refers to the practice of donors choosing to route non-core funding—earmarked for specific sectors, themes, countries, or regions—through multilateral agencies and to the emergence of new multistakeholder initiatives. Drawing on insights from political science and international relations, I put forward an explanation for why these developments are occurring and discuss the consequences for global health research governance.

## Multi-Bi Financing

At first glance, the story of international cooperation in health seems straightforward. Driven by widespread concerns about HIV/AIDS, maternal mortality, and flu pandemics, the past two decades have witnessed an exponential growth in health funding both for service provision, estimated at US$27.73 billion in 2011, and for research, estimated at US$3 billion in 2010 [2],[3]. The growth in funding by governments and international agencies has been accompanied by new forms of multistakeholder cooperation such as the Global Alliance for Improved Nutrition (GAIN) and new institutions including private philanthropists with large endowments such as the Bill & Melinda Gates Foundation.

On the face of it, the rise in funding and plurality of institutions in global health looks like increased support for multilateral cooperation. Existing analyses of global health spending and development assistance that focus on multilateral versus bilateral spending and programs, done by the Institute for Health Metrics and Evaluation and the World Bank, for example, show that over the past 15 years there has been an increase in the budget and commitments of the WHO and World Bank [2]. The WHO program budget has doubled. The World Bank's lending for health has trebled.

Alongside increases in funding for global health, a major change in international cooperation has been the emergence of new multistakeholder institutions such as the Global Fund to Fight AIDS, Tuberculosis and Malaria and the GAVI Alliance. The new initiatives are marked by a structure of governance that differs in five important ways from traditional multilateral institutions (such as the WHO and the World Bank). First, while traditional multilateral institutions are governed by boards solely comprising member states, the Global Fund and GAVI are governed by boards on which sit the representatives of civil society, the private sector, and the Bill & Melinda Gates Foundation. Second, unlike the broad mandates of the WHO (“the attainment by all people of the highest possible level of health”) and the World Bank (“to alleviate poverty and improve quality of life”), both the Global Fund (“to attract and disburse additional resources to prevent and treat HIV/AIDS, TB and malaria”) and GAVI (“to save children's lives and protect people's health by increasing access to immunisation in poor countries”) have narrowly defined mandates that are problem-focused. A third attribute of the new multistakeholder initiatives is that they are entirely funded by voluntary contributions. Fourth, unlike the WHO and World Bank, which work through government agencies and have offices and personnel in recipient countries, neither the Global Fund nor GAVI work directly in-country. Finally, both the Global Fund and GAVI derive their legitimacy from their effectiveness in improving specifically defined health outputs and outcomes in contrast to traditional multilateral agencies, which rely on claims to representation and state-centric deliberation.

But, the story of increasing multilateral funding for global health does not end here. As a recent OECD/DAC report noted, about 40% of the multilateral funding is given through, what it calls, “multi-bi” aid [4]. Changing fastest is the discretionary funding of programs within the WHO and World Bank.

Within WHO, the biennial (2 year) budget has more than doubled in the past decade from US$1.647 billion in 1998–1999 to US$4.227 billion in 2008–2009. Most of the growth, however, has been in extra-budgetary funding, which has risen from 48.8% in 1998–1999 to 77.3% in 2008–2009. In 2007, the top six donors of extra-budgetary funding were US (25%), UK (24%), World Bank-GAVI affiliate (16%), Canada (12%), Bill & Melinda Gates Foundation (11.8%), and Commission of European Communities (10.2%) [5].

**Figure 1:**
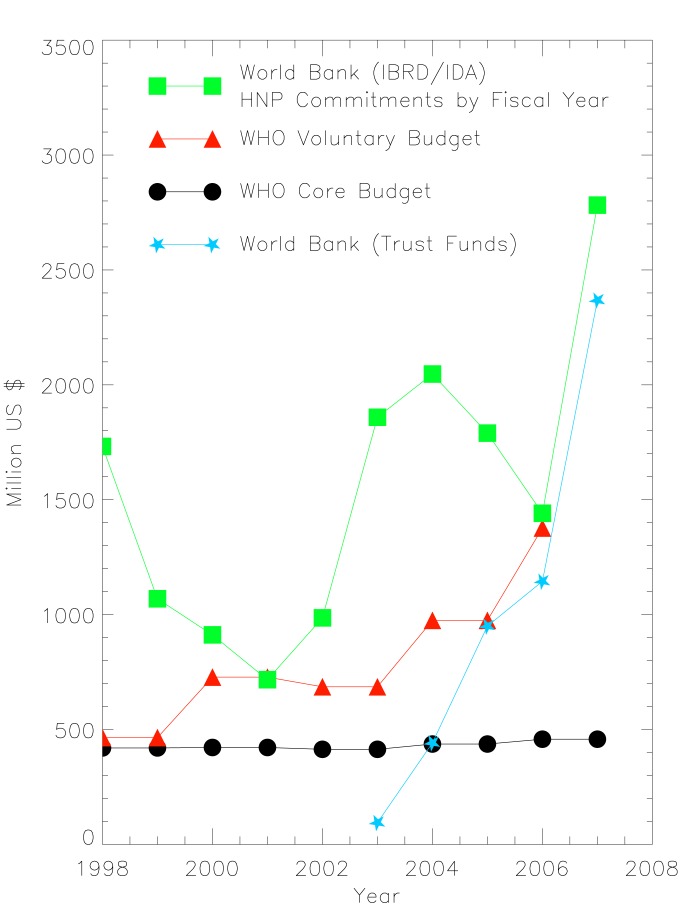
Funding patterns of the WHO and World Bank [5],[6],[11].

Within the World Bank's activities in health, total commitments have increased from US$1.7 billion in 1998–1999 to US$5.2 billion in 2006–2007 [6]. However, a large part of this growth is due to the trust fund portfolio. Trust funds are similar to the voluntary contributions of the WHO in that they are a financing arrangement set up with contributions from one or more donors. A trust fund can be country-specific, regional, or global in its geographic scope, and it can be free-standing or integrated into existing programs.While growth has occurred in both core and trust fund budgets, it is the trust fund portfolio for health that has experienced the most dramatic growth from US$95 million in 2003–2004 to US$2.4 billion in 2006–2007, which is almost equal to the core funding provided through the World Bank's International Bank for Reconstruction and Development and the International Development Association (US$2.8 billion). For both the WHO and World Bank, voluntary contributions are increasing while core budgets are flat or fluctuating (see Figure 1).

## What Is Driving the New Patterns of Global Health Funding and Governance?

The above analysis highlights that an increasing proportion of the new funding for global health has been by contributions that are discretionary (in terms of amount and timing of payment) to fund a specific priority (as opposed to the general purposes of the organization), and to fund implementation through a third party. What explains the shift towards multi-bi financing? It likely reflects a desire by participating governments and other stakeholders such as philanthropic foundations and non governmental organizationsothers, to control and monitor multilateral organizations more tightly.

First, multi-bi financing permits governments and other stakeholders to realign the objectives of multilateral initiatives with their own. Rather than working through the governance of existing organizations, individual governments can use new funding mechanisms as a way to define a separate mandate and to push specific goals.

Second, multi-bi financing permits governments and other stakeholders to tie contributions to performance. For example, the Global Fund and GAVI explicitly link performance to replenishment and must show results to attract donor interest. Similarly, within the WHO and World Bank, negotiations for the amount and time period of voluntary contributions take place outside the official decision-making structures. Thus, through providing tied funding to specific departments, donors can ensure that their funding is used to influence the activities and direction of the organization.

Third, multi-bi financing permits donors to finance and deliver assistance in ways that allow for closer monitoring when they delegate actions to a global fund or agency. One of the main challenges in monitoring the WHO relates to its regional structure, which in practice makes tight policy and budgetary control impossible. In contrast, the Global Fund and GAVI provide detailed financial information about commitments and disbursements, as well as donor pledges and contributions.

## Why Does This Matter for Global Health Research?

There are three possible consequences of multi-bi financing for global health research governance. First is the risk that difficult choices about priority-setting in health will be made in the marketplace of global initiatives, rather than in the community that will have to live with those choices. Developing country health ministers have alleged that this funding mechanism imposes the priorities of powerful states and institutions on poorer countries, whose populations have little recourse to demand accountability or to influence these priorities [1].

As previous work has noted [7]–[10], core funding of WHO is used for the purposes decided by member states at the World Health Assembly while the use of extra-budgetary funding is decided by specific donors. In 2008–2009, of WHO's regular budget, 25% of funds were allocated to infectious disease, 8% to non-communicable diseases, and roughly 4.7% to injuries. These purposes align roughly with the global burden of disease. By contrast, most of the extra-budgetary funding of WHO for 2008–2009 was used for infectious disease (60%), while only 3.9% was for non-communicable diseases and 3.4% for injuries [5].

**Figure 2:**
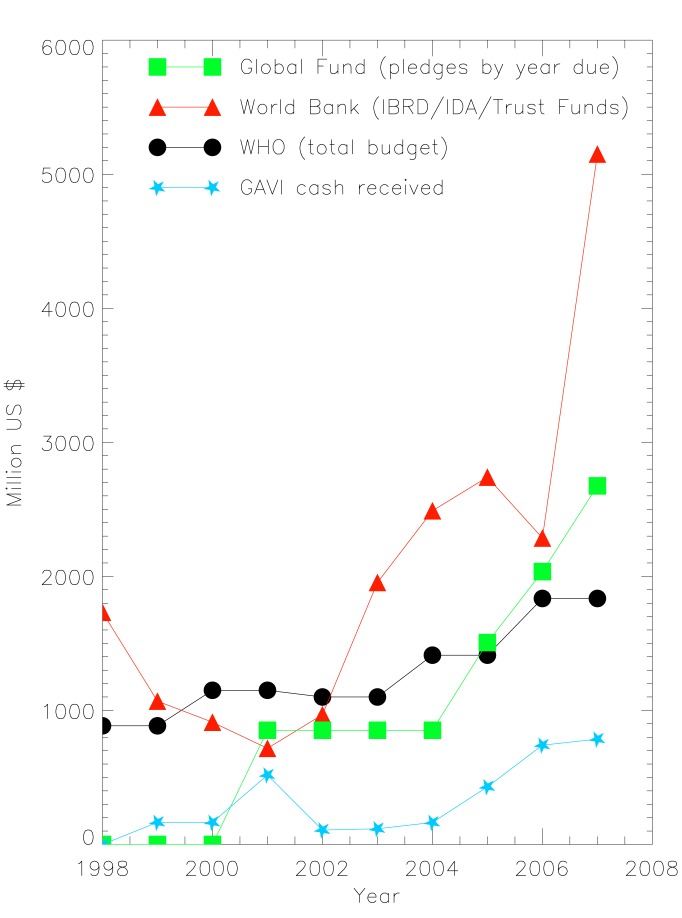
Old and new multilaterals in health [5],[6],[11],[15],[16].

A similar picture emerges at the World Bank. In 2005, the core budget in the health, nutrition, and population sector was focused on infrastructure in health, with the major priorities being health systems (34%), water and sanitation (22%), injury (18%), and disease-specific strategies followed by infectious disease (15%) and non-communicable disease (2%) [5]. In contrast, the trust fund portfolio is largely focused on disease-specific strategies with funds including the Global Partnership to Eradicate Poliomyelitis, Programs for Onchocerciasis Control, the Avian and Human Influenza Facility, and the Global Partnership for TB Control. The Global Fund is the largest trust fund and received 35% of 2008 contributions to trust funds (Figure 2) [11].

Second is the risk that multi-bi financing may create mechanisms that permit donors to favor short-term gains over longer-term public health goals. The advantage of traditional multilateral organizations is that their relative autonomy permits them to bring transparency and discipline to difficult choices: the rationale for creating WHO was to ensure that nations would compromise their short-term differences in order to attain long-term advantages of regularized collaboration and decision-making on health matters. A successful example of this is the International Health Regulations, which require countries to report certain disease outbreaks and public health events. A recent failed attempt was the proposed binding treaty on research and development (R&D) discussed at the World Health Assembly in May 2012. The instrument would have outlined the necessary funding and coordination to promote the R&D that is needed to address the diseases that disproportionately affect developing countries and that constitute a common global responsibility [3],[12].

The third risk is that multi-bi financing will erode global capacities to create, collate, and disseminate information, the cornerstone of research. To some extent, working off the back of several decades of core multilateral funding, donors are still benefitting from a wider, previously built, technical expertise in agencies such as the World Bank or the WHO. However, this knowledge capacity is likely to erode as funding is narrowed to discretionary activities and contributions to core budget are reduced.

But to conclude on a positive note, one major impact of multi-bi financing has been to shine a clear light on how and where multilateral institutions, such as the World Bank and WHO, might do better. Both organizations have been criticized for being slow to act, difficult to monitor, and overly bureaucratic [13],[14]. Multi-bi financing is forcing these institutions to reflect on how to reform to remain more appealing to the wider set of stakeholders and interests at play.
